# Scalability evaluation of a complex community-based falls prevention intervention in Australian stroke rehabilitation

**DOI:** 10.1136/bmjopen-2024-093487

**Published:** 2025-09-25

**Authors:** Ingrid Lin, Sally Day, Catherine M Dean, Lindy Maxted Clemson, Joanne Valentina Glinsky, Anne Cusick, Natasha A Lannin, Katharine Scrivener

**Affiliations:** 1 Department of Health Sciences, Macquarie University, Sydney, New South Wales, Australia; 2 Sydney School of Health Sciences, University of Sydney, Sydney, New South Wales, Australia; 3 Department of Neuroscience, Monash University, Melbourne, Victoria, Australia; 4 Alfred Health, Melbourne, Victoria, Australia

**Keywords:** Stroke, REHABILITATION MEDICINE, Implementation Science, Clinical trials, Randomized Controlled Trial, Behavior

## Abstract

**Abstract:**

**Objectives:**

To investigate the scalability of the multi-component Falls After Stroke Trial (FAST) intervention tailored to community-dwelling adults with stroke to enable post-trial implementation.

**Design:**

A mixed-methods formative evaluation of FAST data guided by the Reach, Effectiveness, Adoption, Implementation and Maintenance (RE-AIM) framework.

**Setting:**

Community settings across three states in Australia.

**Participants:**

Stroke participants were a subset of FAST trial participants (n=50) who were community-dwelling adults who had experienced a stroke up to 5 years prior and were at risk of falling. Therapists who delivered the intervention in the trial (interventionists) were physiotherapists and occupational therapists, trained in the FAST intervention.

**Interventions:**

The FAST intervention is an individually tailored home safety and functional exercise programme designed to reduce falls and improve community mobility. It is offered over a 6-month period using 10 home visits, two telephone calls and programme resources, for example, manual and worksheets.

**Primary and secondary outcome measures:**

Trial data, including interventionist training records and delivery data, resources and stroke participants’ adherence data were used to assess the Adoption, Implementation and Maintenance dimensions of the RE-AIM framework.

**Results:**

The FAST intervention was delivered by 22 interventionists. High implementation fidelity was shown with 90% of the stroke participants receiving FAST dose and content. Effective strategies supporting implementation included standardised programme resources, comprehensive pre-programme training, regular interventionist feedback and interventionist mentoring from experts. Online training and peer support networks will be required for scale up.

**Conclusions:**

This study identifies how a complex intervention to prevent falls after stroke was successfully delivered. The AIM dimensions provided insights to FAST features essential for scale-up. Interventionist training, resources and mentoring/feedback were essential for adoption within the trial. Training and resources should be accessible in an online format for scale up (maintenance).

**Trial registration number:**

ACTRN12619001114134.

**STRENGTHS AND LIMITATIONS OF THIS STUDY**

- Provides an example of early scalability evaluation embedded within a large clinical trial to enable expedited translation into clinical practice.
- Application of a knowledge translation framework (RE-AIM, Reach, Effectiveness, Adoption, Implementation and Maintenance) to inform the content and structure of scalability evaluation.
- Reach data were not collected; effectiveness data was excluded because the trial is ongoing.

## Background

 Translating research interventions into the clinical environment and wider populations is essential but acknowledged to be challenging.^1 2^ This clinical translation, referred to as scaling up, is important to increase reach and impact,^3^ but the challenges in scaling up research interventions are known to be multifaceted.^4 5^ Scaling up a health intervention requires a sequential approach, consideration of contextual and environmental factors and appropriate infrastructure.^6 7^ There are several frameworks which offer guidance on scaling up processes,^7^,^12^ all of which include: outlining the key elements of the intervention (including the process of delivery); and evaluating delivery within the research setting.^13^ Together, evaluation of elements and delivery provide the evidence needed for success in scaling up. However, clinical trialists rarely plan for it,^14^ instead considering scale-up a separate process warranted only after trials of efficacy.^15^ More recently, clinical trialists have been encouraged to evaluate scale-up requirements during the research process so as to understand potential challenges that may be faced.^16 17^ Translation of research knowledge into practice thus requires clinical trialists to embed scalability evaluation early within research studies, particularly for complex interventions.

### A complex falls prevention intervention in stroke

**Figure 1:**
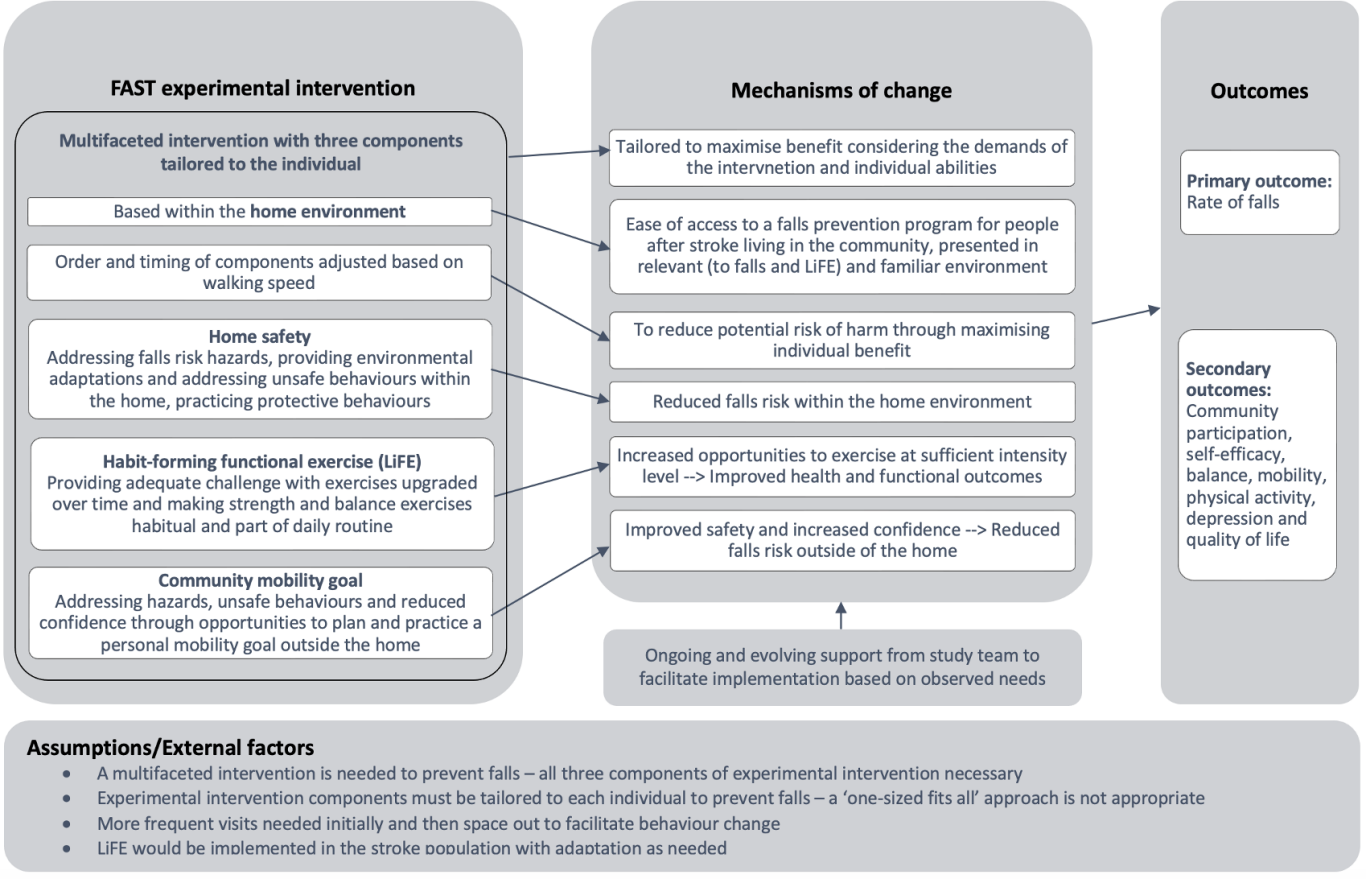
Logic model for the FAST experimental intervention. FAST, Falls After Stroke Trial; LiFE, Lifestyle-integrated Functional Exercise programme.

Falls are a common post-stroke complication.^18^ Post-stroke falls prevention interventions have primarily focused on single components (exercise) with limited effect.^18^ The Falls After Stroke Trial (FAST) (Trial Registration: ACTRN12619001114134)^19^ sought to test a new, multifaceted intervention. The FAST intervention is a complex behavioural intervention aiming to reduce falls in community-dwelling people after stroke. The multiple components of the intervention are tailored to each individual, and a core role of the interventionist is to empower participants to continue the intervention independently—such continuity is known to be difficult.^19^ Scaling up of the FAST intervention will require dedicated planning to enable tailored yet consistent delivery of the multiple programme components in unique home and community environments that can be sustained over the 6 months of programme duration. The intervention was conceptualised by lead research principals Dean (CMD) and Clemson (LMC) with additional development input from team members (KS, SD, NAL and IL). A logic model which illustrates the main causal assumptions of the experimental intervention is presented as figure 1 as per UK Medical Research Council guidance.^20 21^

### Brief description of FAST

The trial protocol for FAST has been published previously,^20^ and the intervention is described briefly here (see online supplemental appendix 1 for the Template for Intervention Description and Replication (TIDieR) checklist). The theoretical context for developing FAST arose because people after stroke fall at up to two times the rate of the general population and experience a higher rate of falls-related injuries.^22 23^ There is also strong evidence that falls after stroke commonly occur in the home environment,^24^ and in Australia, most people after stroke are discharged home.^25^ For this reason, the FAST intervention is home-based.

The FAST intervention includes three components: habit-forming exercise (Lifestyle-integrated Functional Exercise programme, LiFE),^26^ home safety to reduce environmental and behavioural fall hazards,^27^ and support to achieve a community mobility goal (see online supplemental appendix 2). Each addresses different factors known to contribute to falls in people after stroke. The intervention includes balance and lower limb strength exercises and home safety as these interventions prevent falls in older people living in the community.^28 29^ Additionally, practice towards a community mobility goal was included as community participation and outdoor mobility are often limited after stroke due to reduced walking capacity/confidence.^30^

The intervention is delivered by a team, comprising one physiotherapist and one occupational therapist over 6 months via 10 face-to-face visits (7 weekly home visits and three booster visits) and two telephone calls (see online supplemental appendix 3). Within delivery, each component is individualised based on the recipient’s ability, routines, environment and goals, which together can facilitate exercise safety and adherence in people after stroke.^31 32^ In order to maximise the benefit for recipients and account for a range in walking ability, components were introduced in a different order based on the recipient’s walking speed and tailored accordingly. This is because people after a stroke with slower walking speeds are at a greater risk of falls compared with those with faster speeds.^31 33^ Moreover, exercise interventions for people after stroke have been demonstrated to decrease falls in people with faster walking speeds (>0.8 m/s) but increase falls with slower walking speeds (≤0.8 m/s).^31 34^ So it was decided that home safety should be the initial emphasis for recipients with walking speed ≤0.8 m/s.

### Study aims

This paper investigates the scalability of the FAST intervention through a formative evaluation of trial data, including data from the first 50 intervention recipients so as to inform the future adoption of the FAST intervention into clinical practice. Research questions of this study have been structured using three dimensions of the Reach, Effectiveness, Adoption, Implementation and Maintenance (RE-AIM) framework as outlined:

1. Adoption: Who delivered the intervention and what training was required?
2. Implementation: To what extent was the intervention delivered as intended? What strategies were used to support the implementation of the intervention?
3. Maintenance: How could the intervention be adapted for scale up and sustained in the longer term?

## Methods

### Study design

The content and structure of this evaluation were guided by RE-AIM (Reach, Effectiveness, Adoption, Implementation and Maintenance), a framework used for the planning and evaluation of programmes to help translate research into practice.^35^ This study will report only on the AIM dimensions of the framework, as the RE dimensions will be reported in the main trial paper.^36^

#### Patient and public involvement

Patients or the public were not involved in the design, or conduct, or reporting, or dissemination plans of our research.

### Study participants

There were two participant groups to be considered in this scalability evaluation: the people recruited to take part in the trial who received the trial intervention (stroke participants) and the therapists who delivered the trial intervention (interventionists).

#### Stroke participants

A subset of trial participants (the first 50 consecutively randomised participants) allocated to the experimental arm of the trial was included in this study. To be eligible for the clinical trial, people were aged 50 years or older, within 5 years of first stroke, discharged from formal rehabilitation, living in the community, able to walk 10 m across flat ground (with or without a walking aid) and able to provide informed consent. Individuals were excluded if they had moderate-to-severe receptive aphasia (as determined by a score <7/10 on the comprehension component of the Frenchay Screening Aphasia Test)^37^ and/or cognitive impairment (as determined by a score of >4 errors on the Short Portable Mental Status Questionnaire).^38^

#### Interventionists

Interventionists were registered physiotherapists and occupational therapists recruited through professional networks and advertisements. Those interested in delivering the intervention applied and were interviewed to ensure they had availability to deliver the intervention over a 6-month period. Priority was given to therapists with experience in stroke care; however, professionals at all levels of experience were considered and included. All interventionists received FAST training and programme-specific resources.

### Data collection and sources

#### Data about stroke participants and interventionists

Demographic data for stroke participants were collected at baseline (commencement of the trial). Data about the interventionist profession (physiotherapist or occupational therapist) and experience were collected from recruitment records. All participants provided written informed consent for this data to be included.

#### Data on outcomes

Scalability data collection was embedded into the trial design, occurring in parallel with participant recruitment, enrolment, intervention delivery and outcome data collection. Table 1 presents research questions and data sources (units of interest) organised by the adoption and implementation dimensions of RE-AIM.

**Table 1:** Areas of interest based on research questions, units of interest and results for the first 50 trial participants under the adoption and implementation (AI) dimensions

Dimension	Area of interest	Research questions	Units of interest	Results
Adoption	Who delivers the intervention	How many interventionists; what were their characteristics?	Number of interventionists recruited, profession, location	22 interventionists were trained and delivered the intervention to the stroke participants. 12 (55%) were physiotherapists and 10 (45%) were occupational therapists. All had experience working in a community setting/with people after stroke.
	Training – events and resources	What training and educational materials were developed for the interventionists?	Description of initial onboarding training and materials provided	Each interventionist was provided with an intervention manual – including an overview of the intervention, rationale for intervention components, structure of the intervention, delivery of intervention components and trial procedures. Additionally, educational materials (examples of trial resources) – (1) participant folder and copies of the (2) LiFE Trainer’s Manual and (3) LiFE participant’s Manual.^41 42^ A mandatory, 2-day onboarding training session was provided face-to-face for interventionists located in Sydney (86%) or online if interstate (14%). Training consisted of lectures, practical training activities, group discussions, completion of online home safety modules (for occupational therapists), independent review of prescribed literature and programme-specific resources.
	Training – suggestions for improvement	How could the training and resources be improved for the interventionists?	Examples of feedback (from interventionists regarding training) (content analysis)	Positive overall impression of the training – respondents (n=13) described it as ‘well organised’ and ‘extremely thorough’ with ‘comprehensive discussion’. Interventionists expressed that although there was a large amount of content to cover, all of the content presented during training was relevant – ‘I thought all the content was needed’. Interventionists felt that the training made them ‘feel prepared with all aspects [of the intervention)’ and ‘confident and ready to deliver the intervention’.
Implementation	Dose	Were the intervention sessions conducted as often and as long as planned? If not, what were the reasons for this?	Total number and duration of sessions compared with target	45 of the first 50 stroke participants (90%) completed the full 6-month intervention period. On average, the intervention was received by stroke participants as scheduled in terms of the number and duration of sessions (table 2). On average, the physiotherapist conducted a greater proportion of the intervention than the occupational therapist (61% vs 40%).
	Adherence to key components	Were the three intervention components delivered in the intervention sessions as planned?	Number of times each component (home safety, community mobility and habit-forming exercise) was delivered compared with the target	The components of the intervention (online supplemental appendix 2) were received by stroke participants as scheduled (table 2). Minor protocol variations were observed – mainly changes to the timing of session delivery (eg, delayed visits), visit order (eg, varying the order in which the visits were delivered or of components delivered) and mode of delivery (eg, face-to-face to phone delivery). The main reason for variation was the participant/interventionist availability.
	Fidelity – programme completion	How many stroke participants received 100% of the intervention? How many received >80% of the intervention?	Number of stroke participants who received 100% (all 12) and >80% (10 or more) intervention sessions	40 of the first 50 stroke participants (80%) received 100% of the intervention or all scheduled home visits, telephone calls and booster visits. 44 of the first 50 stroke participants (88%) received more than 80% of the intervention. Four stroke participants did not complete the intervention period: non-compliant with intervention (n=2), pain (n=1) and poor health (n=1); one intervention recipient did not start the intervention due to a deterioration in health status.
	Fidelity – delivery	What aspects of the intervention were observed to be delivered as expected? What aspects of the intervention did the interventionists require prompting/feedback on to enable fidelity?	Fidelity tool score, examples of feedback (from fidelity sessions) (content analysis)	Fidelity checks were completed for 13 interventionists who delivered home visits (n=9) and booster visits (n=4). Quality of delivery was largely outstanding (mean (SD) fidelity check score=2.96 out of 3 (0.06)). Interventionists were observed to be familiar with the stroke participants and habit-forming exercise (LiFE), inclusive, prepared and organised. During sessions, trial paperwork was observed to be well used. Habit-forming exercises were actively practiced with stroke participants and home safety recommendations/community mobility goals were reviewed and discussed.
	Engagement	How engaged were the stroke participants with the intervention?	Fidelity tool score (from fidelity sessions) (content analysis)	Intervention recipient engagement with the sessions was scored for 9 of 13 fidelity checks (69%) and was rated mainly as high (89%). Family member or carer engagement was rated where applicable and varied.
	Context	What contextual factors (social and organisational factors and concurrent events) affected implementation?	Description of factors and influence	The COVID-19 pandemic was a significant factor that influenced delivery for 27 of the 45 (60%) stroke participants who completed the intervention. Protocol variations occurred because of face-to-face visits being delivered via telehealth (phone or video call delivery). The majority (63%) of the telehealth visits used telephone calls, while 37% of the visits were completed by video call or a combination. During this period, additional training on safety procedures and telehealth delivery was provided for interventionists. Telehealth was received well by both the interventionists and stroke participants with no apparent change in intervention delivery fidelity, dose or adherence. Many interventionists employed on the trial worked in other roles, which impacted availability for visits and presented an organisational challenge at times.
	Supporting implementation	What strategies were used to support implementation during the intervention period of the trial?	Description of ongoing training, support and facilitation strategies provided, types of resources and training; strategies to support implementation in stroke population	Strategies to standardise implementation and facilitate fidelity were planned and evolved during the intervention period. A summary of strategies to facilitate implementation is in table 3. Implementation strategy terms recommended by the Expert Recommendations for Implementation Change trial for implementation research and practice were used for consistency.^43^ Few and minor strategies were adopted by interventionists to ensure that the intervention was suitable for people after stroke. Additional external cues were occasionally used to prompt stroke participants to perform habit-forming exercises: visual cues (n=6), placement of trial documentation in a visible location (n=2), audio cue (n=1) and prompts from family members and carers (n=6). Regular review of habit-forming exercise principles was required to encourage stroke participants to build a habit and incorporate exercises into their day.

LiFE, Lifestyle-integrated Functional Exercise programme.

#### Adoption

##### Training data

Training data, for example, number and type of training events and feedback were gathered from trial records, research team communications and field notes (kept by research team members).

Data relating to trial resources, including training and education materials, trial team communications, trial records, general and case conference field notes were gathered from the trial records.

### Implementation

#### Dose and adherence data

Data relating to FAST delivery, including the number of intervention sessions completed, number of participants who completed the intervention, duration of intervention sessions, interventionist input, intervention components, protocol variations and intervention adaptations for stroke, were gathered from trial records, research team communications, documentation and field notes.

#### Fidelity and engagement data

Fidelity to the intervention was assessed using a study-specific expert-rated observation of interventionist performance and participant engagement. Intervention fidelity was assessed once per interventionist by a dedicated research team member (expert). Items in the tool were behavioural descriptors of expected performance in key elements to be delivered during the session and were unique to each FAST intervention component. The level of delivery for each key element was rated as omitted (0), could improve (1), well delivered (2) or outstanding (3). Items did not have to be rated if they were not applicable. A total score was derived from summing all available scores and dividing by the number of available scores (range 0–3, higher score indicates better fidelity). Participant engagement (mandatory item) was rated as low, medium or high. Where applicable, written comments providing feedback on delivery from the expert to the interventionist were also included to inform future intervention delivery.

#### Context data

Data relating to contextual factors that affected implementation were gathered from trial records, research team communications, documentation and general and case conference field notes (kept by research team members).

#### Implementation strategies data

Data on strategies used by interventionists to support and facilitate implementation were gathered from feedback surveys, research team communications, trial records and general and case conference field notes.

### Maintenance

Strategies for scaling up and sustaining the intervention over time were derived from adoption and implementation data.

### Data analyses

Quantitative data were collated from units of interest onto an Excel spreadsheet and transferred to IBM SPSS Statistics for Windows, V.27 (IBM, Armonk, New York, USA) for analyses. Descriptive and frequency analyses were completed. Qualitative data, such as interventionist descriptions of experience in training and programme delivery and examples of individualising implementation, were aggregated into descriptive categories by two researchers (KS and IL) identifying similarities and differences in words and phrases. Data were summarised in narrative form.

## Results

### Trial implementation of the experimental intervention

Table 1 presents data for the research questions exploring the adoption and implementation dimensions of RE-AIM. A summary is provided below.

#### Adoption

A cohort of 22 physiotherapists (55%) and occupational therapists (45%) were trained and then delivered FAST to the stroke participants. Most interventionists were experienced, with 64% having more than 10 years of experience and 13% having between 5 and 10 years. However, 23% had less than 5 years of experience. The training was delivered through a 2-day onboarding session with educational materials and FAST resources provided to familiarise interventionists with the three FAST intervention components. They were also introduced to trial procedures, reporting mechanisms and opportunities for mentoring by experts. Feedback obtained for the training process indicated high satisfaction, with the training considered comprehensive and sufficient in preparing the interventionists. See table 1 for further results.

#### Implementation

Most (90%) of the 50 stroke participants (mean age 77 years, time since stroke 1.8 years, baseline walking speed 1.1 m/s; see online supplemental appendix 4 for further baseline characteristics) completed the intervention period, with 80% receiving all 12 sessions. There were minor protocol deviations attributed to participant or interventionist availability, but sessions were delivered and received largely as planned regarding frequency, dose and content (see table 2 for further details). During the COVID-19 pandemic, FAST was delivered via telehealth to more than half (60%) of the stroke participants who completed the intervention. Despite this, fidelity checks revealed the high quality of intervention delivery and participant engagement with strategies such as regular review and reinforcement of the key habit-forming exercise principles, facilitating adherence in the participants. See table 1 for further results.

**Table 2:** Target versus actual number of sessions, duration of sessions and intervention components addressed

	Target	Actual, mean (SD)
Intervention sessions (N=45), n sessions	12	12 (1)
Home visits	7	7 (0)
Telephone calls	2	2 (2)
Booster visits	3	3 (0)
Duration of sessions (N=45), min		
Home visits	60	63 (8)
Telephone calls	30	32 (15)
Booster visits	60	57 (4)
Intervention components for home visits (N=45)		
Baseline walking speed of <0.4 m/s (N=5), n times addressed		
Home safety	3	3 (0)
Community mobility goal	3	3 (0)
Habit-forming functional exercise	4	4 (0)
Baseline walking speed of 0.4–0.8 m/s (N=10), n times addressed		
Home safety	2	2 (0)
Community mobility goal	2	2 (0)
Habit-forming functional exercise	5	5 (0)
Baseline walking speed of >0.8 m/s (N=30), n times addressed		
Home safety	1	1 (0)
Community mobility goal	2	2 (0)
Habit-forming functional exercise	5	5 (0)

Five key strategies (outlined in table 3) were used to support the implementation of the FAST intervention and evolved with identified needs. To ensure effective implementation, (1) comprehensive onboarding training was conducted with additional ongoing training to address identified needs, for example, how to develop a community goal and (2) written educational materials were developed and distributed to interventionists. (3) Clinician implementation team meetings, initially held monthly, offered opportunities for case presentations and discussions, fostering a collaborative learning environment. In addition, (4) feedback loops between interventionists and experts were established via audits of intervention documentation and fidelity visits. Finally, interventionists were encouraged to (5) consult experts on an ongoing basis, via various channels (online platforms and phone communication).

**Table 3:** Recommended strategies to facilitate implementation and scaling up, with format and reason mapped to the adoption, implementation and maintenance (AIM) dimensions

Dimension	Facilitation strategy (trial implementation)	Format delivered in trial	Reason strategy is needed	Maintenance Strategies for scaling up and sustaining the intervention over time
Adoption	Conduct ongoing training	Comprehensive onboarding training with additional training sessions scheduled per-needs if the research team was required to provide further instructions to the interventionists. These additional training sessions were conducted to troubleshoot specific problems relating to implementation of parts of the intervention, for example, completion of trial paperwork, developing a community goal, discussion of common stroke impairments and strategies to manage when completing habit-forming exercise. Six additional training sessions were also scheduled due to changes in trial procedures caused by the COVID-19 pandemic. The additional training sessions covered content relating to telehealth procedures (eg, how to deliver each component via telehealth, equipment set-up and safety tips) and safety procedures when returning to face-to-face visits (eg, screening procedures and use of personal protective equipment).	Plan for and conduct training in an ongoing manner to meet identified areas of need.	The importance of comprehensive onboarding training means it must be included for scale-up. Onboarding training should be adapted to an online format (wider distribution) and include pre-recorded videos and/or online training modules (essential). This should be streamlined (given the large amount of content) for ease of use. Continued access to this is important to enable refresher training. Educational materials (FAST intervention manual, participant folder and LiFE manuals)^38 41^ should be made available on the online platform for download (essential). These should also be reviewed and streamlined for ease of use. The online platform should be monitored by a small team of experts and updated as necessary.
	Develop and distribute educational materials	Programme-specific resources (eg, intervention manuals) were provided to interventionists at onboarding to introduce them to the intervention and delivery procedures. Various written materials (eg, visit schedule cheat sheets, a stroke supplement, home safety toolkits, examples of community goals, guidelines on COVID-19 safety procedures for interventionists to review; and home safety handouts, community mobility checklists for interventionists to provide to stroke participants) were developed/collated by research team members through continuing review of processes and feedback. These were made available to interventionists as deemed necessary.	Develop and distribute supporting materials to interventionists to support intervention delivery. Interventionists required supporting materials to deliver the intervention.	
Implementation	Organise clinician implementation team meetings	This was given regularly in the form of case conferences, completed over Microsoft Teams. Case conferences were attended by interventionists and one to several trial team members to enhance feedback and mentoring. During case conferences, the trial team would provide a brief update on the trial, one to two cases would be presented by interventionists with time allocated for discussion and questions, and the trial team would present on particular aspects of the intervention if necessary (eg, developing relevant community mobility goals and/or in the GAS format, review of how to complete assessments and paperwork). Case conferences were conducted on weeknight evenings, generally over a 1-hour period. They were recorded to allow those who could not attend to view the recordings. Case conferences were initially conducted monthly and became less frequent over time.	Provide time and a collaborative learning environment to promote information-sharing and problem solving.	As individual mentoring is not sustainable, general support should be provided on the online platform and include a Frequently Asked Questions section which addresses common queries, a section with downloadable FAST-specific resources and a case studies section which provides real-life examples of the FAST intervention being delivered to people after stroke. As individual mentoring is not sustainable, the fidelity tool should be made available for download to be used as a self-reflection tool or feedback tool (eg, during a mentoring visit involving two interventionists). The online platform should include peer-support groups or forums to facilitate discussions among interventionists (to enable individualised support, provision of feedback/information sharing and promote self-management). A small group of experts should be available to provide brief feedback, eg, via a contact function/email on the online platform (though this may not be monitored regularly).
	Provide ongoing consultation	Interventionists could make regular contact throughout the week with research team members to report progress and troubleshoot any issues (eg, to discuss visit scheduling, to discuss purchasing of home safety equipment, to brainstorm ideas for community mobility goals and exercises). Research team members were also consulted as needed for specific advice. Contact occurred primarily through Microsoft Teams, although email and phone communication were also used.	Regular communication between the trial team and interventionists (ongoing mentoring/feedback loop) to support implementing the intervention.	
	Audit and provide feedback	One member of the research team would audit intervention documentation and provide feedback to interventionists weekly. Written feedback and support (eg, what was done well, reminders if elements were missed, suggestions for/reminders of components to be covered in the next session) were provided through posts and comments on Microsoft Teams. Verbal and written feedback was also provided during fidelity visits where a research team member conducted a joint visit with an interventionist and reviewed key elements of intervention delivery. One fidelity visit per interventionist was planned.	To monitor, evaluate and modify interventionist behaviour. To provide interventionists with feedback on intervention delivery.	

FAST, Falls After Stroke Trial; GAS, Goal Attainment Scale; LiFE, Lifestyle-integrated Functional Exercise programme.

### Adapting the experimental intervention for scale-up

Table 3 presents data exploring the maintenance dimension of RE-AIM.

#### Maintenance

To scale up and sustain the FAST intervention over time, results indicate the following strategies will be useful. (1) Interventionist training, FAST education materials (used in interventionist training) and programme resources (used by interventionists and stroke participants) need to be available on an online platform for easy access. Interventionist training needs to incorporate appropriate learning approaches to retain elements of instruction, familiarisation with resources, exercise practice and expert/peer feedback and FAST educational materials and programme resources need to be available online with an ‘option to print’ to enhance availability and cater for a diverse range of user situations. Further, (2) streamlining of training content, FAST educational materials and programme resources will reduce the volume of material to be navigated and enhance user experience by highlighting components most used. Lastly, (3) scale-up will involve a shift away from individualised expert fidelity assessment, feedback and expert-facilitated peer-support groups which were time-intensive and resource-intensive and not realistic in scale-up, towards self-assessment and self-reflection modules embedded into interventionist education materials. A FAST online platform could also provide a mechanism for community of practice peer-to-peer sharing and support and ideally have the local and/or online leadership of ‘super-users’ who could provide individualised support which was valued by interventionists, and act as champions for awareness and uptake in diverse settings.

## Discussion

FAST delivered a complex intervention, with its mechanism of action grounded in theory and empirical evidence from prior research. The results of this evaluation demonstrate that the FAST intervention was adopted well and implemented efficiently and with fidelity, indicating the ability to scale up at trial completion.^39^

Key elements needed to maintain the programme in scale-up were identified with successful adoption underpinned by trial design choices and specific strategies. In the first instance, interventionists were registered health professionals, specifically physiotherapists and occupational therapists, with existing specialised expertise in programme content and mechanisms. Second, interventionists were given comprehensive and structured initial training, programme resources and access to support from FAST intervention experts. Third, interventionists had considerable autonomy around the timing of programme delivery (within specified targets) and sequencing of the content of the programme so they could tailor it to stroke participant needs, goals and abilities. Fourth, interventionist reporting processes meant that they were continually in contact with and accountable to the research team across the delivery of the programme.

Trial implementation was per-protocol with stroke participants receiving the set number and content of FAST sessions. Delivery mode amendments had to be made for some stroke participants who were recruited during the COVID-19 pandemic. For these stroke participants, face-to-face visits were not possible and the same content and strategies were implemented using telehealth approaches. Implementation of the FAST intervention was thus feasible, and it had sufficient flexibility to permit adaptation of session timing, sequencing and use of different components (habit-forming exercise, home safety and the community mobility goal) to meet unique stroke participant needs and abilities as well as contextual challenges. Successful implementation of the FAST intervention within the trial required substantial time and resources and was conducted in a metropolitan setting. Therefore, setting-dependent factors must be carefully considered when planning implementation in other contexts. For broader scale-up, funding mechanisms will also need to be addressed. In the Australian context, several options may support programme delivery, including a fee-for-service model (eg, Commonwealth government-funded home care packages and private health insurance) or integration within existing services such as community health programmes.

There are three primary considerations regarding the scalability of FAST. First, to maintain successful implementation with more interventionists in more diverse locations, access to and availability of training, educational materials and programme resources needs to be direct, without the necessity of expert facilitation by experienced clinicians. These could be primarily distributed via an online platform, and ideally there would be no pay-wall barrier to access the materials. Second, maintaining the consistency and quality of FAST content and dose (number of sessions) would benefit from streamlining the presentation of educational materials and programme resources would enhance user experience. In scale-up, individualised support from a FAST expert would not be available, so the FAST online platform should provide the entry point, onboarding, navigation and access framework for a seamless user experience. The simplest modification could be using evaluation data from the study to identify the most used FAST components and highlight these for easy access while making less used components available but in an archive area. Other modifications to enhance user experience would be determined by the functionality of any online platform but could include self-directed learning modules, Frequently Asked Questions and navigation guides. Further, case studies from trial implementation could be included in educational materials and programme resources to support the development of FAST intervention-specific clinical reasoning skills. However, it is important to note that updating and maintaining online materials can be costly and notoriously difficult to sustain.

Third, maintenance of mentoring to support interventionists throughout implementation was identified to be important. In the trial, FAST experts engaged in a continuous feedback loop with interventionists, providing individualised feedback to enable protocol adherence and cumulative development of intervention implementation expertise and facilitating active reflection on mechanisms of action embedded in the intervention that meant they put theory into practice. Fidelity or coaching tools were used and are increasingly being seen as useful in implementing and disseminating evidence-based interventions as they are intended. When used as either self-report or by someone with experience or by both, they can provide helpful therapist insights and improved fidelity and participant responsiveness.^40^ Therefore, in scale-up, opportunities for self-assessment, self-reflection and peer support remain critical and online methods and prompts could be trialled within an online platform or direct email methods.

This study had both strengths and limitations. FAST has a robust programme logic design grounded in theory and informed by empirical evidence, where individual components have been shown to be effective and when combined, offer a tailored and targeted intervention to reduce falls in a known at-risk population. The study also drew on multiple data sources and included robust data on programme delivery, aligned to a knowledge translation framework. However, the study would have been strengthened by the inclusion of all aspects of the RE-AIM framework alongside qualitative perspectives to provide deeper insights into experiences and contextual factors influencing implementation.

## Conclusion

This opportunity to reflect on how FAST was operationalised within a trial setting has provided data of use for future scale-up. Specifically, we have sought to understand how the intervention was adopted and implemented per-protocol with embedded flexibility and had an opportunity to understand contextual challenges faced by interventionists. This evaluation revealed FAST content and dose as delivered by registered physiotherapists and occupational therapists in paired teams and experienced by stroke participants can be maintained without alteration in any scale up. Modifications are likely needed in terms of access to and availability of educational materials and programme resources, training and the assurance processes used for fidelity and continuing quality improvement in service provided by interventionists. These modifications do not require a change to the programme per se—rather modifications are to expand access to and ease of use for existing materials and shift expert-facilitated support to online self-directed learning and peer-support strategies. With these modifications to the delivery of resources, training and support and with knowledge of the outcome of the full clinical trial when it is available, the FAST programme will be ready for large-scale uptake.

## Supplementary material

10.1136/bmjopen-2024-093487 online supplemental appendix 1

## Data Availability

Data are available upon reasonable request.
