# Supplementary material for: Scalability evaluation of a complex community-based falls prevention intervention in Australian stroke rehabilitation
Source: BMJ Open. 2025 Sep 25;15(9):e093487. doi: 10.1136/bmjopen-2024-093487 (PMC12481250; doi:10.1136/bmjopen-2024-093487)
Supplement: online supplemental appendix 1 [file bmjopen-15-9-s001.docx]

**Appendix 1. TIDieR checklist describing the experimental and control interventions**

| **Checklist item** | **Experimental intervention** | **Control intervention** |
| --- | --- | --- |
| Brief name | **Falls After Stroke Trial (FAST) intervention**: habit-forming functional exercise (LiFE), home safety and community mobility | Usual care (no active intervention) |
| Why | People after stroke experience varied physical and cognitive impairments, resulting in a high rate of falls, particularly in the home environment. It is hypothesised that a multifaceted and tailored intervention, involving the person and their environment, is required to prevent falls. The exercise component of the intervention is novel in that it aims to embed new habits in daily life and involves behaviour change. | Pragmatic trial design |
| What | All participants receive all three intervention components of FAST; however, each component is introduced based on the participant’s baseline walking speed. The amount and timing of the three components also vary based on baseline walking speed. | Participants may continue to participate in their usual care which may or may not include some general exercise training |
| Who provided | An occupational therapist and physiotherapist work as a team to deliver FAST. All therapists are trained in delivering the habit-forming exercise and community mobility components. The occupational therapists deliver the home safety component. |  |
| How | Most FAST is delivered face-to-face during home/booster visits, with a few sessions conducted over the phone. |  |
| Where | The therapist conducts the intervention at the participant’s home. Community mobility may involve visits to a community venue or use of transport if relevant to the participant’s goal. |  |
| When and how much | Each participant receives ten home visits (seven consecutive and three booster sessions) and two phone calls over six months. Most home visits occur over the first seven weeks, with weekly visits completed at this time. |  |
| Tailoring | Each intervention component has individualised plans developed with the therapist and the participant. Habit-forming exercises are chosen and completed based on the participant’s ability and daily routines, home safety recommendations are individualised to the participant and their environment, and the community mobility goal is planned with each participant in a GAS format. |  |
| Modifications | Modifications will be documented throughout the intervention period and reported at the end of the trial. |  |
| How well | Fidelity checks of intervention sessions will be completed using fidelity tools designed specifically for each intervention component. All fidelity tools include observations based on key elements for therapist and participant receipt and enactment of the intervention. This will be reported at the end of the trial. |  |

**Appendix 2. Overview of experimental intervention components**

| **Component** | **Rationale** | **Content** |
| --- | --- | --- |
| Home safety | The home environment may present hazards that lead to falls in people after stroke. Falls in people after stroke commonly occur in the home environment (1) and in Australia, most people after stroke are discharged home.(2) Addressing these fall hazards, providing environmental adaptations and addressing unsafe behaviours may reduce falls risk within the home. Further, practising negotiating hazards rather than elimination was encouraged in the more physically able. | Occupational therapist assessment of participant’s home – fall hazards and protective behaviours.  Recommendations to improve safety are provided.  Simple home modifications up to AUD$200 per participant (e.g. provision of equipment).  More complex home modifications require referral to community occupational therapy services. |
| Community mobility goal | People after stroke are less likely to access the community compared to healthy older adults and are at a higher risk of falls when accessing the community.(3) This leads to social isolation and reduced physical activity. Additionally, hazards, unsafe behaviours and reduced confidence may lead to falls outside of the home and in the community in people after stroke. Addressing these factors and providing opportunities to practice mobility outside of the home/in the community may improve safety and confidence and reduce falls risk. | Occupational therapist or physiotherapist assessment of mobility/community access.  Strategies to mobilise safety outside of the home/in the community were discussed.  Strategies to increase opportunities to access the community discussed.  Goal to improve mobility/community access created on the Goal Attainment Scale (GAS).(4) |
| Habit-forming functional exercise (LiFE) | This intervention component comprises the Lifestyle-integrated Functional Exercise program (LiFE), which is efficacious in reducing falls in older adults living in the community.(5) LiFE involves balance and strengthening exercises which are completed at an appropriate level of challenge for each participant and upgraded over time. LiFE is embedded into the participant’s daily routine so that the completion of exercises becomes habitual. In theory, habituating exercise behaviour should increase exercise opportunities, thus improving health and physical outcomes. | Occupational therapist or physiotherapist assessment of the participant’s daily routine and ability using LiFE-specific assessment tools.  Opportunities to incorporate strength and balance exercises into specific daily tasks established and are upgraded over the intervention period. |

References:

1. Kerse N, Parag V, Feigin VL, et al. Falls after stroke: results from the Auckland Regional Community Stroke (ARCOS) Study, 2002 to 2003. Stroke. 2008;39(6):1890-3.

2. Senes S. *How we manage stroke in Australia*. Australian Institute of Health and Welfare; 2006. Accessed January 8, 2024. <https://www.aihw.gov.au/getmedia/65ff1dda-b514-4c56-8ea9-740e479a72c7/hmsa.pdf.aspx?inline=true>

3. McCluskey A, Ada L, Kelly PJ, et al. Compliance with Australian stroke guideline recommendations for outdoor mobility and transport training by post-inpatient rehabilitation services: an observational cohort study. *BMC Health Serv Res*. 2015;15(1):1-9.

4. Turner-Stokes L. Goal attainment scaling (GAS) in rehabilitation: a practical guide. Clin Rehabil. 2009;23(4):362-70.

5. Clemson L, Singh MAF, Bundy A, Cumming RG, Manollaras K, O’Loughlin P, et al. Integration of balance and strength training into daily life activity to reduce rate of falls in older people (the LiFE study): randomised parallel trial. BMJ. 2012;345:e4547.

**Appendix 3. Schedule of the experimental intervention**

| **Intervention week** | **Visit** | **Slow Walker**  **< 0.4 m/s** | **Medium Walker**  **0.4-0.8 m/s** | **Fast Walker**  **> 0.8 m/s** |
| --- | --- | --- | --- | --- |
| **Week 1** | HV1 | Introduction  Home safety assessment and planning | Introduction  Home safety assessment and planning | Introduction  Habit-forming exercise assessment and planning |
| **Week 2** | HV2 | Home safety | Habit-forming exercise assessment and planning | Habit-forming exercise |
| **Week 3** | HV3 | Habit-forming exercise assessment and planning | Habit-forming exercise | Home safety assessment and planning |
| **Week 4** | HV4 | Habit-forming exercise | Habit-forming exercise | Habit-forming exercise |
| **Week 5** | HV5 | Home safety | Home safety | Home safety |
| **Week 6** | HV6 | Habit-forming exercise | Habit-forming exercise | Habit-forming exercise |
| **Week 7** | HV7 | Habit-forming exercise | Habit-forming exercise | Habit-forming exercise |
| **Week 9** | TC1 | Phone call | Phone call | Phone call |
| **Week 13** | BV1 | Booster visit | Booster visit | Booster visit |
| **Week 15** | BV2 | Booster visit | Booster visit | Booster visit |
| **Week 19** | TC2 | Phone call | Phone call | Phone call |
| **Week 23** | BV3 | Booster visit  Final assessment | Booster visit  Final assessment | Booster visit  Final assessment |

HV = Home visit; TC = Telephone call; BV = Booster visit

**Appendix 4. Table of baseline characteristics of stroke participants**

| **Characteristic** | **N** | **Mean (SD) or N (%)** |
| --- | --- | --- |
| Location  Sydney  Canberra | 50 | 48 (96%)  2 (4%) |
| Age, years | 50 | 77 (11) |
| Gender, female | 50 | 20 (40%) |
| Time since first stroke, years | 50 | 1.8 (1.1) |
| Time since formal rehabilitation, years | 29 | 1.4 (1.0) |
| Baseline walking speed, normal (m/s) | 50 | 1.1 (0.5) |
| Baseline walking speed, n participants enrolled  < 0.4 m/s (slow)  0.4-0.8 m/s (medium)  > 0.8 m/s (fast) | 50 | 5 (10%)  11 (22%)  34 (68%) |
