# Supplementary material for: Scalability evaluation of a complex community-based falls prevention intervention in Australian stroke rehabilitation
Source: BMJ Open. 2025 Sep 25;15(9):e093487. doi: 10.1136/bmjopen-2024-093487 (PMC12481250; doi:10.1136/bmjopen-2024-093487)
Supplement: Supplementary file 2 [file bmjopen-2024-093487.reviewer_comments.pdf]

PEER REVIEW HISTORY

BMJ Open publishes all reviews undertaken for accepted manuscripts. Reviewers are asked to complete a checklist review form and are provided with free text boxes to elaborate on their assessment. These free text comments are reproduced below.

ARTICLE DETAILS

Title (Provisional)

Scalability evaluation of a complex community-based falls prevention intervention in Australian stroke rehabilitation

Authors

Lin, Ingrid; Day, Sally; Dean, Catherine; Clemson, Lindy Maxted; Glinsky, Joanne Valentina; Cusick, Anne; Lannin, Natasha A; Scrivener, Katharine

---

VERSION 1 - REVIEW

**Reviewer**	**1**
**Name**	**Saywell, Nicola**
**Affiliation**	**Auckland University of Technology**
**Date**	**19-Mar-2025**
**COI**	**None**

Thank you for the opportunity to review your work. It is a thoughtful, comprehensive and well written evaluation, and the intervention has the potential to reduce falls in people with stroke in the community.

Methods

Interventionists

It would be good to note if any inclusion criteria were placed on the interventionists. Could they be new grads, or those with minimal neurological physiotherapy experience this would increase scalability considerably? The reason I am not sure is that at the start of the discussion, prior to the information about FAST training the interventionalists were described, “with existing specialised expertise in program content and mechanisms”. Was this an inclusion criterion for becoming an interventionalist?

Discussion

It is a great aspiration to scale up interventions and doing it early in the trial is the perfect time. There is so much in this paper which has been done well but the acknowledgement of some of the key barriers is missing. I have outlined a couple which I believe would need to be overcome.

1. An inclusion criterion for the participants recruited to this trial was that they were not currently receiving any treatment. These participants would have received inpatient rehabilitation and any outpatient or community rehabilitation as per usual care. What I think

needs to at least be mentioned in the discussion is how the FAST intervention might be funded, when the majority of developed countries struggle to provide any community rehabilitation immediately after discharge from hospital. I can see that a reduction in injurious falls could render this intervention cost-effective (and any reduction in falls will reduce suffering and pain) but you have not looked at cost-effectiveness. This may be a hard sell to any government funding agency and grant funding is difficult to gain for a project with no endpoint.

2. Maintenance and updating of online material are expensive and notoriously difficult to sustain. The job frequently falls on universities and researchers and tends to fall over when the "champion" leaves or moves to another role. Online platforms also frequently stop being supported and become obsolete, which then causes a huge amount of work to move material to ensure it is still available.

**Reviewer**	**2**
**Name**	**Patton, Susan**
**Affiliation**	**University of Arkansas**
**Date**	**06-Jul-2025**
**COI**	**None**

Thank you for providing an opportunity to review your manuscript. The study investigating the scalability of the FAST intervention using data from the FAST intervention and guided by the RE-AIM framework makes a significant contribution to the science of all prevention in the post stroke community dwelling population. There are many strengths to your report - the introduction and background show context of post-stroke falls as well as scaling of health interventions. The literature is well referenced and relevant. The research questions are well defined and fill a knowledge gap. The methods are described with sufficient detail to replicate and the results and conclusions are well stated and linked to the original research questions. Presenting the research questions, data elements, and results in a Table makes them understandable. There are two items that I believe you should consider adding. One is addressing research ethics. I feel that this was part of the intervention study TIDieR and maybe a statement to that effect is what is needed. Second, what are the strengths and limitations of the study?

VERSION 1 - AUTHOR RESPONSE

Reviewer: 1

Interventionists

It would be good to note if any inclusion criteria were placed on the interventionists. Could they be new grads, or those with minimal neurological physiotherapy experience this would increase scalability considerably? The reason I am not sure is that at the start of the discussion, prior to the information about FAST training the interventionalists were described, "with existing specialised expertise in program content and mechanisms". Was this

an inclusion criterion for becoming an interventionalist?

Thank you for raising. The following text has been added to the methods to clarify:

"Priority was given to therapists with experience in stroke care; however, professionals at all levels of experience were considered and included."

Additionally, the following data has been added to the results to clarify experience levels:

"Most interventionists were experienced, with 64% having more than 10 years of experience and 13% having between 5 and 10 years. However, 23% had less than 5 years of experience."

Discussion

It is a great aspiration to scale up interventions and doing it early in the trial is the perfect time. There is so much in this paper which has been done well but the acknowledgement of some of the key barriers is missing. I have outlined a couple which I believe would need to be overcome.

1. An inclusion criterion for the participants recruited to this trial was that they were not currently receiving any treatment. These participants would have received inpatient rehabilitation and any outpatient or community rehabilitation as per usual care. What I think needs to at least be mentioned in the discussion is how the FAST intervention might be funded, when the majority of developed countries struggle to provide any community rehabilitation immediately after discharge from hospital. I can see that a reduction in injurious falls could render this intervention cost-effective (and any reduction in falls will reduce suffering and pain) but you have not looked at cost-effectiveness. This may be a hard sell to any government funding agency and grant funding is difficult to gain for a project with no endpoint.

Thank you, a good consideration. The discussion has been modified and now reads:

"Successful implementation of the FAST intervention within the trial required substantial time and resources and was conducted in a metropolitan setting. Therefore, setting-dependent factors must be carefully considered when planning implementation in other contexts. For broader scale-up, funding mechanisms will also need to be addressed. In the Australian context, several options may support program delivery, including a fee-for-service model (e.g., Commonwealth Government-funded home care packages and private health insurance) or integration within existing services such as community health programs."

2. Maintenance and updating of online material are expensive and notoriously difficult to sustain. The job frequently falls on universities and researchers and tends to fall over when the "champion" leaves or moves to another role. Online platforms also frequently stop being supported and become obsolete, which then causes a huge amount of work to move material to ensure it is still available.

The following text has been added to the relevant paragraph in the discussion:

"However, it is important to note that updating and maintaining online materials can be costly and notoriously difficult to sustain."

Reviewer: 2

One is addressing research ethics. I feel that this was part of the intervention study TIDieR and maybe a statement to that effect is what is needed.

A statement has been added as requested, thank you for identifying this oversite.

"The Falls After Stroke Trial (FAST) (Trial Registration: ACTRN12619001114134; Macquarie University Ethics Approval Numbers: 5201500456 and 520231547551507)"

Second, what are the strengths and limitations of the study?

The following paragraph has been added as requested by the reviewer.

NOTE to editor: We have previously provided the strengths and limitations dot points, we will be guided by you if you also wish for the following paragraph to be included.

"This study had both strengths and limitations. FAST has a robust program logic design grounded in theory and informed by empirical evidence, where individual components have been shown to be effective, and when combined, offer a tailored and targeted intervention to reduce falls in a known at-risk population. The study also drew on multiple data sources and included robust data on program delivery, aligned to a knowledge translation framework. However, the study would have been strengthened by the inclusion of all aspects of the RE-AIM framework alongside qualitative perspectives to provide deeper insights into experiences and contextual factors influencing implementation."
